# Informal STEM Learning for Young Children: A Systematic Literature Review

**DOI:** 10.3390/ijerph19148299

**Published:** 2022-07-07

**Authors:** Suzanne Alexandre, Yaoying Xu, Melissa Washington-Nortey, Chinchih Chen

**Affiliations:** 1 Department of Counseling and Special Education, Virginia Commonwealth University, Richmond, VA 23284, USA; alexandresg@vcu.edu (S.A.); ccchen@vcu.edu (C.C.); 2 Department of Psychology, King’s College, London SE1 1UL, UK; melissa.washington-nortey@kcl.ac.uk

**Keywords:** Informal STEM, dual language learners, school readiness, social/emotional development, social/emotional learning

## Abstract

Studies show that children spend considerable time engaged in informal learning outside of educational settings. Informal educational settings such as museums can provide a variety of opportunities to engage children in STEM learning and scientific discovery, which can increase school readiness. Research has also determined an achievement gap in students from low socio-economic backgrounds and in students who are dual language learners. The literature shows that this gap begins even before children enter formal schooling. This systematic review serves two purposes: to explore the impact of informal STEM learning (ISL) on school readiness and to examine the relationship between ISL and children’s social-emotional development, particularly in children who are dual language learners. Using PRISMA procedures, we identified 36 eligible studies in this systematic review. The findings illuminate the important role of parents and/or caregivers and the quality of design and interventions used at ISL sites, such as museums and zoos, on how ISL can impact school readiness for preschoolers. No research was found to specifically address the impact of ISL on school readiness for children who are dual language learners. The implications from the findings suggest that further research is needed on ISL for young children, particularly considering the dearth in research on young dual language learners. The implications further suggest that parents, ISL site designers, facilitators, and educators can benefit from learning about the impact of ISL on school readiness.

## 1. Background

Preschool children spend more than 80% of their waking hours engaging in informal learning experiences outside of school settings [1,2,3,4,5]. Informal educational settings such as museums provide a variety of opportunities to engage children in STEM learning and scientific discovery [2,6,7]. Researchers have also found that participation in informal science activities fosters children’s abilities of scientific reasoning and increases their commitment to science learning [2,8].

School readiness is the set of skills that prepares children for later school success. It signifies a child’s ability to meet school and classroom expectations related to age-appropriate cognitive, language, and social skills [9]. The key domains of school readiness include language, literacy, cognition and general knowledge, approaches to learning, physical health (e.g., well-being and motor development), and social and emotional development (e.g., self-regulation and social skills) [10,11,12]. Data across the U.S. suggest that many preschool children are not meeting these milestones at kindergarten entry. For example, the results of the Virginia Kindergarten Readiness Project [11] suggest that 34% of children in Virginia arrive at kindergarten without necessary preparation to be successful in one or more critical learning domains (literacy, math, self-regulation, and social skills).

Among children who are dual language learners (DLL), meaning students for whom English is not their primary language, 48% were considered unprepared when entering kindergarten. Data also show that children who enter kindergarten behind rarely catch up; instead, the achievement gap tends to widen over time [11,13], particularly for children who are from culturally and/or linguistically diverse backgrounds. Among these children, young Latinx DLLs are the largest growing population, with an estimated 13% of the U.S. population coming from Spanish-speaking homes [10,14]. Among over 460 languages represented in U.S. schools and programs, the majority (about 77%) of DLLs speak Spanish as their first language [15,16]. Unfortunately, the achievement gap between Spanish-speaking DLLs from low-income families and their peers is already evident prior to kindergarten entry [17,18,19,20] and continues at every level of education [21,22]. Given the critical role of the preschool period and the increasing number of Spanish speakers in the U.S., there is an urgent need to help these children prepare for school prior to their entry to kindergarten.

Young children’s socio-emotional competence affects both school readiness and future outcomes. Children with emotional or behavioral problems are more likely to have poorer academic outcomes in the areas of language and numeracy performance [23,24,25,26]. Extant evidence also shows that socio-emotional competence predicts future academic achievement [24,26]. Studies that have examined the influence of both school readiness and socio-emotional competence on academic achievement suggest that socio-emotional competence may have stronger predictive impacts on academic achievement [27]. These findings highlight the importance of socio-emotional competencies for predicting school readiness and other social and academic outcomes. Socio-emotional competence is highly related to social interactions, which require that children explain their thinking, ideas, and problem-solving process through peer interaction and interacting with adults. Informal learning often involves such context with explanations to enhance children’s learning by deepening their own understanding of the problem and seeking solutions to the problem [28,29,30]. Social interactions within informal learning can engage children in sustained learning built upon their own interests and initiatives [31,32,33]. Through informal learning, the child freely chooses how and what they engage with, motivated by their curiosity.

Despite the literature documenting the importance of informal learning for young children, little is known about the impact of ISL on young children’s school readiness, particularly their social-emotional skills. The purposes of this systematic review are two-faceted. First, we intend to explore the overall impact of ISL on preschool-aged children’s school readiness, or the enhancement of STEM skills for school-age children. Second, we further examine the relationship between ISL and children’s social-emotional development, particularly children who are dual language learners. The following research questions are addressed:

1. What are the effects of informal STEM learning on school readiness?
2. What is the impact of informal STEM learning on children’s social-emotional development?
  (a) Does informal STEM learning have a different impact on children who are dual language learners?

## 2. Method

### 2.1. Search Strategy

PRISMA procedures were used to identify studies regarding the impact of ISL on school readiness, and more specifically on children’s social-emotional learning. The databases used in the search included ERIC (Education Resources Information Center), All ProQuest, Education Research Complete and PsycInfo (American Psychological Association). In order to ascertain the strongest query terms, an initial search was undertaken in the varying databases using synonyms for the words preschool, school readiness, informal learning, and the “STEM” acronym and related terms. A title and abstract scan of each article on the first page of the results that these initial searches brought about were scanned for relevance. Additionally, the lists of keywords were examined to determine which synonyms of the search concepts came up most frequently. Through this process it was determined that several terms were needed to encompass the “preschool child” concept; therefore, the terms preschooler, preschool, preschool children, preschool kid, pre-school children, and young child were selected. A similar process was followed with the concept of school readiness and it was determined that this term on its own, rather than any synonyms of it, was the best fit for the search string.

As a result of the synonym and keyword searches, the final search string included the following terms: preschooler, preschool children, preschool kid, pre-school children, young child, and school readiness. Due to the complexity of the STEM acronym in terms of its breadth and the need for the studies to be specific to informal settings, it was determined that the best approach for capturing the five STEM components and/or any combination of the components and informal learning was to use an adjacency string. The following strand was created and used in addition to the terms previously mentioned: (informal OR “informal learning” OR museum) N5 (STEM OR science OR technology OR mathematics OR math OR maths OR engineering). The time frame from 2010 to August 2021 was used as a field quantifier in each database while running the aforementioned search string.

### 2.2. Inclusion and Exclusion Criteria

Studies were included based on four inclusion criteria pertaining to the research questions. One, they were studies that investigated ISL and/or any investigation of an individual or combination of the STEM components in an informal setting. Studies were excluded if they focused on formal STEM learning. Two, they included preschoolers and/or children in the range of three to eleven years old. Studies of elementary school children were included due to the lack of research on informal STEM that focused specifically on preschoolers. Three, research was empirical and published by peer-reviewed journals. Literature reviews and technical reports were excluded. Four, studies were included only if they were written in English.

### 2.3. Screening and Coding Procedures

The results of the initial database search (n = 977) were uploaded to Zotero for further screening. A scan for duplicates was conducted using the Zotero software. The duplicates (n = 151) were removed, leaving 826 articles. A title and abstract scan of each remaining study was then conducted to determine if the study was a good fit based on the inclusion and exclusion criteria. This process eliminated 784 studies. One study was necessarily eliminated after the title and abstract scan due to it being unavailable. The remaining 42 studies were subjected to a full review to determine if they could contribute to answering the two research questions. A detailed coding sheet was created to organize the studies during the process of the final text scan. The coding sheet included the following categories: author, title, abstract, publication, year of publication, keywords, DOI, code, reviewer, independent variable/s, dependent variable/s, theoretical framework, sample size, student demographics, research design, unit of analysis, method of analysis, main findings, implications, and limitations. 

**Figure 1:**
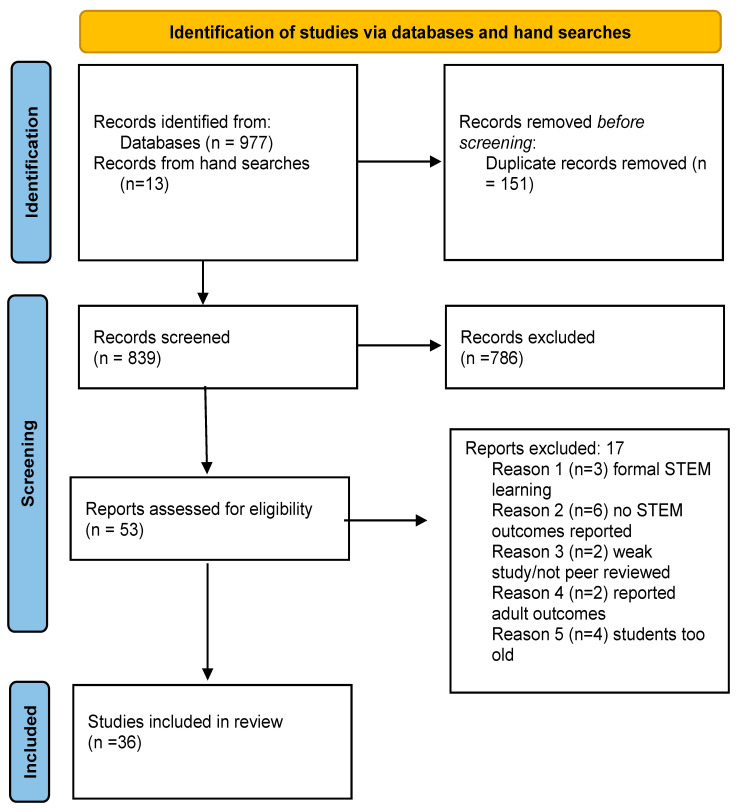
Study selection process.

The full screening process excluded 17 studies. Reasons for the exclusions were as follows: studies (n = 3) pertained to formal as opposed to ISL, studies (n = 6) did not include STEM outcomes, one study was weak in general with no sample size and limited demographic information, one study was not peer-reviewed, two studies reported adult outcomes, and in one study the students were too old, beyond the P-5 grade level inclusion criterion. The reference lists of included studies were examined to determine if any relevant and important studies had been overlooked through the searching process. Thirteen studies were identified through this hand searching method, resulting in 36 studies for inclusion in this review. See Figure 1.

## 3. Results

### 3.1. Characteristics of ISL Research

The participant demographics, research design, and findings of the studies used in this review are summarized in Table 1, Table 2, and Table 3, respectively. Of the studies included in this review, 22 used quantitative methods, 9 used qualitative methods, and 6 used a mixed methods approach. Among the studies that used quantitative methods, there was wide variation in the number of participants ranging from 23 to 2163. The studies that used qualitative methods had a range in number of participants. Three of the qualitative studies had only one participant [34,35,36], while the largest sample in the qualitative studies had 392 participants [37]. The studies that implemented a mixed methods approach ranged in the number of participants from 32 to 199.

The reported gender of participants in the samples was approximately evenly distributed. Of the studies that reported on racial demographics, the participants were diverse and typically numbered in the highest percentage of the students being White, then Black, then Latinx, then other. Nine of the studies reported on the socio-economic status of the participants [37,38,39,40,41,42,43,44,45].

Sixteen of the reviewed studies used children as the unit of analysis. Seventeen studies used another common unit of analysis, caregiver and child. Four of the studies [1,39,41,46] used educator and child as the units of analysis, and two studies [44,45] used only the caregiver as the unit of analysis. 

There were three main types of sites for ISL that appeared in the studies: children’s homes, afterschool programs and informal stem learning sites (ISLS) such as museums, zoos, aquariums, and botanical gardens. The majority of reviewed studies (n = 23) occurred in ISLS. The number of studies that examined ISL in a child’s home environment was 10. There were three studies [39,47,48] that explored afterschool programs. One study [49] included home environments and museums, and one [50] included all three site types. Field trips were researched in two of the studies [51,52]. The following is a description of the studies that relate to the research questions in each of these types of informal learning sites.

**Table 1:** Participant demographics.

Author(s)	Title	Participants
Acosta et al. (2021) [53]	Whether and how knowledge moderates linkages between parent–child conversations and children’s reflections about tinkering in a children’s museum	111, 5–10 yo; 60 male, 51 female; 48 White, 25 Latino, 12 Black, 4 Asian, 6 mixed; majority of parents had a bachelor’s degree or higher
Alexander et al. (2012) [49]	Longitudinal analysis of the relations between opportunities to learn about science and the development of interests related to science	215, 4 yo, 86 White, 6 Black, 3 Latinx,, very small % Asian or Native American
Allen et al. (2019) [47]	From quality to outcomes: a national study of afterschool STEM programming	1599, youth 4th–12 grade, 45% female, 25% Black, 14% Latinx, 30% White, 2% Native, 3% Asian
Andrews and Wang (2019) [34]	Young children’s emergent science competencies in everyday family contexts: A case study	One 7 year old female
Booth et al. (2020) [54]	Parents’ causal talk: links to children’s causal stance and emerging scientific literacy	153, 3 yo, 71 boys; 27.5% of mothers had no higher than high school education; 13.1% African American, 73.9% White, 2.6% Asian, 10.5% multiple races
Callanan et al. (2017) [38]	Family science talk in museums: predicting children’s engagement from variations in talk and activity	83, 3–11 yo, 40 boys, ethnically diverse, majority middle to upper class; years of parents’ schooling 12–24 years
Carol-Ann Burke (2020) [39]	Informal science educators and children in a low-income community describe how children relate to out-of-school science education	32, 9–14 yo, from low SES families, 23 instructors, 2 exhibit developers, 11 community leaders
Chung et al. (2019) [40]	Quick response code scanning for children’s informal learning	91 youth mean age 8.54, 43 female, 36 male, SES median income $53K
Eberbach and Crowley (2017) [7]	From seeing to observing: how parents and children learn to see science in a botanical garden	79, 6–10 yo, 49 girls, 30 boys, 90% white, 6% Asian, 4% Black, 92% of parents had college degree
Ehsan et al. (2021) [55]	Computational thinking embedded in engineering design: capturing computational thinking of children in an informal engineering design activity	10, 5–7 yo, 8 boys, 2 Black, 3 multiracial, 5 White
Gold et al. (2021) [41]	Engineering play with blocks as an informal learning context for executive function and planning	110 preschoolers, 44% female; 25% children with disabilities, majority had low SES
Goldstein et al. (2019) [50]	Researching a new pathway for promoting children’s active outdoor science exploration in urban settings	81, 6–9 yo and 34 parents
Gomes and Fleer (2019) [35]	The development of a scientific motive: how preschool science and home play reciprocally contribute to science learning	One 4 yo old boy, both parents have tertiary college degrees
Haden et al. (2014) [31]	Supporting family conversations and children’s STEM learning in a children’s museum	130, 4–8 yo, 61 girls; 71 White, 33 black, 26 Hispanic, 87% of parents w/college degree
Hightower et al. (2021) [56]	Maybe we do more science than I had initially thought’: How parental efficacy affects preschool-aged children’s science and math activities and media use	199 parents of 3–5 yo, 8 White, 2 Black, 1 Asian, 1 Latino; all had received education beyond high school
Joy et al. (2021) [57]	Understanding parents’ roles in children’s learning and engagement in informal science learning sites	63, 3–18 yo, 60.3% female, 31 families and 44 parents
Kızıltaş and Sak (2018) [51]	Integrating field-trip activities with other activities in the preschool curriculum: its effects on the preschoolers’ social–emotional skills	36, 4–5 yo Turkish children, exp group: 10 girls, 8 boys; control group: 9 girls, 9 boys
Katz (2011) [36]	A case study of the use of internet photobook technology to enhance early childhood “scientist” identity	one 6 yo boy, interviewed again at 8 yo
Kisiel et al. (2012) [58]	Evidence for family engagement in scientific reasoning at interactive animal exhibits	41 families with 3–17 yo, 77.3% of parents had post-secondary schooling
Kornelaki and Plakitsi (2018) [1]	Thunderbolt hunt. Educational program for students from 5 to 9 years old in the archaeological museum of Ioannina	136, 5–8 yo and 12 teachers, 3 classes from private schools, 5 from public schools
Leonard et al. (2016) [48]	Social justice, place, and equitable science education: broadening urban students’ opportunities to learn	33, 8–12 yo, urban students, 8 Black, 22 LatinX
Leyva et al. (2021) [42]	Relations between subdomains of home math activities and corresponding math skills in 4-year-old children	78, 4 yo and their parents, mostly middle-income and White
Luisa et al. (2021) [37]	Children’s protagonism in a science exhibition: an exploratory study of an exhibition in Rio de Janeiro (Brazil)	392, 5–8 yo children from a low SES community
Marcus et al. (2017) [59]	STEM Learning and transfer in a children’s museum and beyond	40, 5–6 yo and their mothers, 20 male, 45% White, 25% Asian, 12.5% Black, 7.5% Hispanic, 2.5% Middle Eastern, 7.5% mixed; mean level of maternal education: 16.31 years
Marcus et al. (2018) [60]	Promoting children’s learning and transfer across informal science, technology, engineering, and mathematics learning experiences	64, 4–8 yo, 67% white, 11% Black, 9% Asian, 5% mixed race, 80% of mothers had a Bachelor’s degree or higher, 63% of fathers had a Bachelors degree or higher
Morais (2015) [61]	Storytelling with chemistry and related hands-on activities: informal learning experiences to prevent “chemophobia” and promote young children’s scientific literacy	29, 8–10 yo
Mulvey et al. (2020) [62]	Interest and learning in informal science learning sites: differences in experiences with different types of educators	979 children (409 early childhood, 378 middle, 215 adolescent), 59.8% female, 60.9% White; 1184 adults 72.6% female, 71.2% White
Pagano et al. (2019) [63]	Conversational reflections about tinkering experiences in a children’s museum	248 family groups 6–11 yo
Pattison et al. (2020) [64]	Understanding early childhood engineering interest development as a family-level systems phenomenon: findings from the head start on engineering project	15 families with preschool children, 8 families who reported speaking Spanish at home
Plummer and Small (2018) [52]	Using a planetarium fieldtrip to engage young children in three-dimensional earning through representations, patterns, and lunar phenomena	46, 6–7 yo, 23 boys
Ramani et al. (2015) [65]	Math talk during informal learning activities in Head Start families	33, 3–5 yo, 60% female; all enrolled in Head Start; 39% ESL; 67% Black, 12% Hispanic, 12% mixed, 9% white; 33% speak English and another language
Schellinger et al. (2019) [43]	Using technology-enhanced inquiry-based instruction to foster the development of elementary students’ views on the nature of science	129, 4th and 5th graders, approx half male, 43 low SES
Strawhacker and Bers (2018) [46]	Promoting positive technological development in a kindergarten makerspace: a qualitative case study	20, 5–7 yo 67% White, 11% Black, 6% Hispanic, 9% Asian, 5% mixed; 80% of the children’s mothers and 63% of fathers held a bachelor’s degree or higher
Vandermaas-Peeler et al. (2016) [66]	Parent guidance of young children’s scientific and mathematical reasoning in a science museum	23, 4–6 yo and their families, 13 girls
Willard et al. (2019) [67]	Explain this, explore that: a study of parent–child interaction in a children’s museum	65, 4–6 yo, 30 girls, 35 boys, 47 White, 7 Hispanic, 4 Asian, 1 Black
Zhang et al. (2020) [16]	Parent/child number application activities predict children’s math trajectories from preschool to primary school	196, 5 yo from 20 preschools in Guangdong province in south China; 95 boys and 101 girls
Zheng and Libertus (2021) [44]	Individual differences in parental support for numeracy and literacy in early childhood	259 parents of 3–6 yo, 13% of parents had a high school diploma or less, 39% had a bachelor’s degree or higher, mean income $60K
Zucker et al. (2021) [45]	Expectancy-value theory & preschool parental involvement in informal STEM learning	208, 3–5 yo, mostly middle class, 70% of parents had a bachelor’s degree or higher

**Table 2:** Summary of research designs.

Author(s)	Research Design
Acosta et al. (2021) [53]	Quantitative design-based approach
Alexander et al. (2012) [49]	Quantitative, prospective longitudinal, correlational
47. Allen et al. (2019) [47]	Quantitative
Andrews and Wang (2019) [34]	Qualitative case study
Booth et al. (2020) [54]	Quantitative
Callanan et al. (2017) [38]	Quantitative design based research, quasi-experimental
Carol-Ann Burke (2020) [39]	Multi-methods
Chung et al. (2019) [40]	Quasi-experimental and mixed methods; design-based
Eberbach and Crowley (2017) [7]	Quantitative quasi-experimental
Ehsan et al. (2021) [55]	Qualitative case study
Gold et al. (2021) [41]	Quantitative correlational
Goldstein et al. (2019) [50]	Mixed methods
Gomes and Fleer (2019) [35]	Qualitative case study
Haden et al. (2014) [31]	Quantitative experimental
Hightower et al. (2021) [56]	Exploratory sequential mixed methods
Joy et al. (2021) [57]	Quantitative descriptive
Kızıltaş and Sak (2018) [51]	Quantitative experimental, static group pre/post test design
Katz (2011) [36]	Qualitative
Kisiel et al. (2012) [58]	Qualitative case study
Kornelaki and Plakitsi (2018) [1]	Mixed methods
Leonard et al. (2016) [48]	Mixed-methods, quasi-experimental
Leyva et al. (2021) [42]	Quantitative
Luisa et al. (2021) [37]	Qualitative
Marcus et al. (2017) [59]	Quantitative experimental
Marcus et al. (2018) [60]	Quantitative experimental
Morais (2015) [61]	Qualitative, content analysis
Mulvey et al. (2020) [62]	Quantitative
Pagano et al. (2019) [63]	Quantitative, comparative
Pattison et al. (2020) [64]	Qualitative case study
Plummer and Small (2018) [52]	Mixed methods
Ramani et al. (2015) [65]	Quantitative
Schellinger et al. (2019) [43]	Quantitative
Strawhacker and Bers (2018) [46]	Qualitative ethnographic case study
Vandermaas-Peeler et al. (2016) [66]	Quantitative experimental
Willard et al. (2019) [67]	Quantitative experimental
Zhang et al. (2020) [16]	Quantitative correlational
Zheng and Libertus (2021) [44]	Quantitative correlational
Zucker et al. (2021) [45]	Quantitative

**Table 3:** Summary of findings of studies.

Author(s)	Main Findings
Acosta et al. (2021) [53]	Parent STEM talk during experience increased child’s STEM talk during and after the experience; if a child had prior experience, and/or received orientation, more STEM talk occurred during tinkering and reflections
Alexander et al. (2012) [49]	Early science interests were strong predictors of later opportunities to engage in ISL, whereas the opposite pattern (early opportunities predicting later science interests) was not found
Allen et al. (2019) [47]	Increases in STEM engagement, identity, career interest, career knowledge, relationships, critical thinking, and perseverance; largest gains when engaging with activities for 4 weeks or more; higher-quality programming led to more growth
Andrews and Wang (2019) [34]	Child’s emergent science competencies were playful with a developing understanding of NOS; family learning included spontaneous and purposeful learning; mother’s scaffolding played important role
Booth et al. (2020) [54]	The higher the degree to which parents talk about causally relevant information, the stronger is the child’s causal stance; the higher the degree to which parents invite the child to generate their own explanations, the more advanced their scientific literacy will be
Callanan et al. (2017) [38]	With priming experience, children’s engaged talk was strongly predicted by the frequency of parents’ critical thinking questions; children of more elaborative parents seemed to learn more in a museum exhibit; asking questions may encourage children’s engagement but providing explanations may reduce children’s engaged talk
Carol-Ann Burke (2020) [39]	Educators underestimated level of interest children had in ISL and the range of home ISL activities in which children were participating; intentional and repeated hiding of the word science can communicate to child that ISL is reserved for more elite social groups
Chung et al. (2019) [40]	Effective ISL tools promote interest; QR code scanning was effective in promoting knowledge gains; ISL can be used to support increased future learning
Eberbach and Crowley (2017) [7]	When families engaged in more disciplinary talk during experience, children were more likely to learn from it; simple training was sufficient to improve parent disciplinary talk
Ehsan et al. (2021) [55]	K-2 children are capable of engaging in computational thinking (CT) when designing a solution to an engineering problem; engineering design can be appropriate and promising context for practicing CT
Gold et al. (2021) [41]	Fostering young children’s early engineering thinking using play might improve other learning and cognitive domains and overall school readiness
Goldstein et al. (2019) [50]	Toolkit promotes urban youth and families’ participation and engagement with science concepts and practices across a range of informal, outdoor contexts; this likely relates to educator and parent support
Gomes and Fleer (2019) [35]	Parents often do not have an understanding of how children can learn science in play; home play experiences have rich possibilities that together with the preschool activities can contribute to developing a scientific motive
Haden et al. (2014) [31]	Adults who received conversation instructions asked more Wh-type questions; adults in the Inspector Sturdy Build + Talk group produced more STEM-related talk; families who received building tip had highest ratio of braces-to-total-pieces; children who received building instructions mentioned more types of STEM related content in photo-narrative task
Hightower et al. (2021) [56]	Parental perceived efficacy in supporting child’s early STEM learning is related to the number of related informal activities their children engage in
Joy et al. (2021) [57]	Parents’ requests for science information and interactive exhibits may be important factors for learning behaviors in children; when parents asked more questions, children more likely to observe exhibit; if exhibit was not interactive, children more likely to provide science explanations and less likely to engage with exhibit
Kızıltaş and Sak (2018) [51]	Pretest scores of groups were not significantly different; posttest scores showed positive effect of field-trip activities on social–emotional skills of children in experimental group; follow-up test 12 weeks later found positive effects persisted
Katz (2011) [36]	Children’s play activities can lead into established science
Kisiel et al. (2012) [58]	In ISL contexts, observation and interaction play an important role in engaging visitors in practices of scientific argumentation and reasoning
Kornelaki and Plakitsi (2018) [1]	Learning community influences learning process and science education
Leonard et al. (2016) [48]	Participants’ science knowledge increased significantly in both settings; interacting with actual artifacts helped students anchor learning to activities and develop specific science knowledge
Leyva et al. (2021) [42]	Home math activities in some subdomains (i.e., adding/subtracting, set comparison, and patterning) were aligned with children’s corresponding competences, but others were not
Luisa et al. (2021) [37]	Strategies used by mediators can directly affect children’s behavior; mediation overrides design of exhibition when it comes to children’s experiences, highlighting importance of training for explainers
Marcus et al. (2017) [59]	Dyads in engineering information group used more pieces to brace the structures; EIG had a higher ratio of functional pieces to total pieces; EI did not lead to differences in frequency of STEM talk; exception was children’s technology talk, which was more frequent for control group
Marcus et al. (2018) [60]	Engineering instructions, either alone or in combination with transfer instructions led to use of engineering principle; ETI was linked to some differences across groups in parents’ and children’s STEM talk; parents’ STEM talk varied with child age
Morais (2015) [61]	The hands-on activities and storytelling may engage students through listening, reading, imagining, understanding, making, and explaining, and thus can generate interest in science and scientific research
Mulvey et al. (2020) [62]	Visitors who interacted with youth or adult educators believed they learned more, reported more interest in topics, and showed improved content knowledge over those who interacted just with the exhibit
Pagano et al. (2019) [63]	The most detailed reflections occurred among families who participated in the program with a design challenge; families who had creations with them during reflections elaborated more; children who had combined experience of tinkering with design challenge and reminiscing with their creation demonstrated highest levels of STEM talk
Pattison et al. (2020) [64]	Evidence of critical shifts in parent awareness, knowledge and values; family re-engagement with engineering activities; and increased family use of engineering design process
Plummer and Small(2018) [52]	Students developed more sophisticated three-dimensional learning as they participated in planetarium field trip experience and classroom instruction
Ramani et al. (2015) [64]	The frequency of engaging in number-related activities at home predicted children’s foundational numerical knowledge; quality of math talk used while engaging in number activities predicted children’s advanced numerical knowledge
Schellinger et al. (2019) [43]	Technology-rich, inquiry instruction across formal and informal settings can shape elementary students’ views of some aspects of NOS
Strawhacker and Bers (2018) [46]	Children engaged in most Positive Technological Development (PTD) aspects, but showed somewhat less evidence of collaboration and community building; the space demonstrated support in most areas except for community building
Vandermaas-Peeler et al. (2016) [66]	Parents in instruction group provided elaborated guidance to enhance children’s evaluations of experiments, and their children responded with increased accuracy
Willard et al. (2019) [67]	Instructional intervention to parents influenced interactions with child; parents’ behavior affected how children engage with exhibit; children’s ability to understand and recreate gear machines on own predicted by previous interactions with parent in a gear exhibit
Zhang et al. (2020) [16]	Frequency of parent–child formal math activities not associated with children’s math trajectories; frequency of informal math activities was associated with math skill levels in preschool; parental involvement in application activities during preschool years predicted rate of growth in formal math skills through first grade
Zheng and Libertus (2021) [44]	SES variables were related to active literacy activities, whereas few SES differences were seen in parents’ numeracy activities; several domain-specific associations between parental beliefs and enrichment activities were seen
Zucker et al. (2021) [45]	Parents do not engage in STEM daily with their preschoolers, even when considering simple activities such as counting or describing the weather; parents who feel empowered to do science and math engage their preschooler in informal STEM learning more often

### 3.2. Effects of ISL on School Readiness by ISL Types

The majority of the studies (n = 34) included in this review address the question of the effects of ISL on school readiness and/or the enhancement of STEM skills. For the purpose of this review, “the enhancement of STEM skills” refers to the studies that address how STEM skills are impacted by ISL, but in that the participants are already school age. A total of nine studies included in this review studied children who were preschool age, eighteen studied children who were school age, and eight of the studies included participants who were in preschool and elementary school. Two of the studies address neither school readiness nor how ISL enhances STEM skills [39,49]. Rather, Alexander et al. [49] studied the relationship between parent reports of children’s interests related to science learning and the opportunities they had for science learning. Carol-Ann Burke [39] examined how informal science educators and children describe the attitudes, dispositions, and experiences that influence the informal science education practices of children. Factors that are found to impact school readiness at each of the three main site types are explained below.

When the home environment was examined as a place for ISL, there were several factors found to be influential. One factor that contributes to STEM gains at home is the opportunity for investigative play [16,34,35,65]. A caregiver’s STEM talk, questioning and scaffolding for the child is another important factor for school readiness [34,42,65]. Several of the studies suggest that guidance for caregivers toward ISL activities can foster STEM skills in children [42,45,50,56,64,65]. A child’s interest in STEM can also influence school readiness [35,53,64]. Additionally, several studies explored caregivers’ beliefs about STEM learning and their own self-efficacy with STEM concepts as elements that influence children’s school readiness [42,44,45,56,64].

Upon examination of afterschool programs, several studies found that they do contribute to the enhancement of STEM skills [39,47,48,50]. Two factors attributed to the success of afterschool programs for enhancing STEM learning are the duration of the program and the quality of the program [47,48]. Investing in afterschool programs also contributes to positive student outcomes [47]. The amount of training and support for staff and other adults who help with the afterschool programs is also a factor in outcomes for students [39,50]. Positive STEM outcomes were also reported when afterschool programs created meaningful content connections for students, such as opportunities to interact with actual artifacts [16,39,50].

Informal learning at sites such as museums, zoos, aquariums, and botanical gardens significantly enhanced children’s STEM learning. When participants, including caregivers and children, were prepared with information and scaffolding before entering the exhibit, they reported positive learning outcomes [7,31,38,53,59,60,66,67]. The frequency and quality of caregivers’ and staff’ guiding questions and causal talk during participation in the exhibit also resulted in positive outcomes for children [1,7,37,38,53,54,57]. When children were given the opportunity to both observe and interact with the exhibit, they reported positive learning results [1,41,55,57,58,61,62]. The opportunity to talk and reflect after leaving the exhibit was also shown to positively influence STEM outcomes [53,63]. Staff/educator training was reported as being beneficial to STEM outcomes for children in ISLS [1,37,62].

Informal learning on field trips enhanced STEM learning and interest and helped to develop children’s social-emotional skills [51,52]. Researchers found that field trips aided first grade students in developing more sophisticated science content knowledge when the field trip was combined with formal instruction [52]. The findings of Kızıltaş and Sak [51] demonstrated that when preschool students participated in field trips complementing their curriculum, their social-emotional skills were significantly positively affected as compared to students who received classroom instruction only.

### 3.3. Impact of ISL on Social-Emotional Development

There were three studies that addressed the impact of ISL on social-emotional development [46,47,51]. Allen et al. [47] carried out a quantitative study to examine the impact of afterschool programs on social emotional learning. The sample included 1599 youths (grades 4–12) enrolled in 158 STEM-focused afterschool programs across 11 states. The study reflects a positive impact of the programs on social-emotional development. Most youth (65–85%) reported increases in STEM engagement, identity, career interest, career knowledge, relationships, critical thinking, and perseverance. Results also suggest that both the duration (at least 4 weeks) and quality of the program positively impacted students. Kızıltaş and Sak [51] conducted an experimental study of 136 5 to 8 year-olds to determine if field trips in addition to classroom learning could strengthen students’ social-emotional development. They found that students in the experimental group (field trip experience in addition to classroom instruction) reported a significant positive effect on social-emotional skills. A follow-up twelve weeks later found that the positive effects from the field trip experience persisted. Strawhacker and Bers [46] examined how participation in technology activities through makerspace can make positive contributions to social development. They found that the majority of the children showed gains with application of the Positive Technological Development (PTD) framework. Some of the social-emotional skills addressed by this framework are communication, collaboration, community building, and choices of conduct.

### 3.4. Impact of ISL on Children Who Are Dual Language Learners

A very limited number of studies (n = 2) were found that address ISL and dual language learners [64,65]. Neither of the studies address the impact of ISL on dual language learners specifically, rather they were listed in the demographic descriptions of the samples. It is evident that there is much need for research in this area.

## 4. Discussion

### 4.1. Inquiry Themes Emerging from This Review

There were some commonalities among the research questions in the studies that were reviewed. Although some of the research questions that surfaced were outliers, three main inquiry themes emerged from the coding system of included articles: (1) parent/child STEM interactions at home, (2) parent/child/staff interactions at ISLS and their impact on STEM learning, or more specifically their impact on school readiness, and (3) if and how ISL impacts children’s social emotional learning. Questions around the first theme of parental involvement in STEM activities at home arose several times. These questions addressed whether parents’ talk related to a child’s early STEM literacy, the relationship between a child’s STEM interest and/or identity and STEM opportunities at home, and how playful contexts contribute to children’s development of STEM concepts. Researchers were also interested in making connections between parents’ education levels, socioeconomic status and perceived self-efficacy in STEM concepts and how these impacted a child’s STEM readiness and motivation. Questions around the second theme addressed adult/child interactions at ISLS, such as whether these interactions impact a child’s museum experience in terms of learning outcomes and engagement with the exhibit, how participation in an ISLS experience would mediate science education, and how playful contexts at an ISLS contribute to children’s development of STEM concepts. The third inquiry theme relates to if and how ISL impacts a child’s social emotional learning in the context of field trips, makerspace, and after-school programs. These three themes help address the research questions of this systematic review.

### 4.2. Relationship between ISL and School Readiness/Early STEM

Findings of this systematic review confirm the importance of informal learning for children’s STEM knowledge and skills development, which aligns with the existing literature (e.g., [2,7]). The current study also identified the sites of ISL programs that involve home, afterschool programs, and community-based programs. This further supports the existing literature, particularly to emphasize the critical role of family involvement. However, despite the recognition that home environment and parent facilitation play an important role in engaging children’s STEM learning, only 25% (9/36) of the studies focused on preschool children, with very limited information on the impact of ISL on school readiness. Additionally, only 5.6% (2/36) included children who were DLLs, despite the fact that young Latinx DLLs are the largest growing population in the U.S. [10,14]. While ISL contributes to children’s overall development and learning, additional research is needed to explore its specific impact on children’s school readiness, particularly children from culturally and linguistically diverse backgrounds. In relationship to the inquiry theme of caregiver/child interactions at home and at ILS and the findings that demonstrate the importance of such interactions, there is obviously a disconnection between what is available to support children and their families who have access to resources and what is missing for children and families who are under-resourced, which ironically but not surprisingly confirms the achievement gap that has been persistent for decades [13].

### 4.3. Relationship between ISL and Social-Emotional Development

Social-emotional development is one of the identified predictors for children’s future academic achievement and overall success [27]. Findings of this systematic review suggest positive impacts of ISL on children’s social-emotional development. However, very few studies focused on preschool-aged children, although social-emotional development is one of the key domains in school readiness. On one hand, educators and researchers recognized that young children spend more than 80% of their waking time in informal learning and through social interaction in informal learning children develop social emotional skills. On the other hand, the majority of studies on ISL focused on school-aged children when a gap had started even before kindergarten entry. This seems to be a self-conflicting rationale: We propose to prepare children to be ready for school to reduce the achievement gap, yet we would not measure them until they are at school age. As a result, the achievement gap persistently exists and even widens over time. Instead of retrospectively identifying the problem, what is needed is to develop preventative programs *before* they enter kindergarten and document the long-term impact of early ISL on their later school success.

### 4.4. Research Implications

The findings of this systematic review suggest that there is a need for more research on ISL for young children. The majority of the studies that have been done attend to school readiness in the academic sense and/or the impact of ISL on children’s interest in STEM. Very little research has addressed the impact of informal learning on preschool children’s social-emotional competence and overall school readiness. More empirical studies in this area could benefit researchers, educators, museum educational staff, and parents. Likewise, there is a dearth of research regarding the impact of ISL on young dual language learners. Research in this area could be of benefit to this population in helping stakeholders understand how best to prepare preschool dual language learners for formal schooling.

Findings of this review have important implications for possible interventions with components that empower parents and/or caregivers to engage their children in meaningful science learning activities. Parents in our reviewed studies identified doing many science activities with their children once they were shown examples of what constituted informal science learning. Therefore, it is important to make informal science learning explicit and relevant to parents, particularly parents from different cultural backgrounds. In addition to qualitative studies identifying parents’ perceptions and attitudes, studies using experimental controls will contribute to the existing literature with empirical data.

### 4.5. Practical Implications

Early childhood educators can benefit from the findings of this systematic review by incorporating ISL in their ongoing professional development, particularly through engagement of children and parents in the process of afterschool program development and implementation. Findings of the current review suggest that children’s math experiences in the home environment can help their numerical knowledge development, which will transfer to formal school learning. It is essential to establish a school-home-community partnership to ensure learning occurs in all authentic settings. Findings of this study strongly suggest that informal learning environments are important factors to consider in science education for children of all ages. Social justice and learning environments are intertwined and should be clearly demonstrated as an outcome of equitable science teaching and practice. Educators and parents can work together to design home or community-based afterschool programs with meaningful learning opportunities for children and their families to explore informal science learning.

## 5. Conclusions

The intended contribution of this study is to influence research on informal STEM learning to better prepare students for formal schooling for the purpose of reducing the achievement gap of students who are DLLs. This systematic review confirms the importance of informal learning for children’s STEM knowledge and skills development, as supported by the existing literature (e.g., [2,7]). The current study further identified the critical role of family involvement in children’s STEM knowledge and skills development, which leads to school readiness and future success. However, limited studies focused on preschool children, with very limited information on the impact of informal STEM learning on school readiness. Additionally, only a couple of studies in this systematic review included DLL children, despite the fact that young Latinx DLLs are the largest growing population in the U.S. Further research is needed to explore the specific impact of informal STEM learning on children’s school readiness, particularly preschool-aged children from culturally and linguistically diverse backgrounds. Given the importance of informal learning for young children and the critical role of family involvement, empirical research is needed to examine the effects of family involvement on young children’s STEM knowledge and skills development across all authentic settings.
